# Supplementary material for: Analyses of Fatty Acids, Proteins, Ascorbic Acid, Bioactive Phenolic Compounds and Antioxidant Activity of Canadian Barley Cultivars and Elite Germplasm
Source: Molecules. 2022 Nov 14;27(22):7852. doi: 10.3390/molecules27227852 (PMC9693253; doi:10.3390/molecules27227852)
Supplement: Supplementary file 1 [file molecules-27-07852-s001.zip › molecules-2013155-Supplementary.pdf]

Table S1: Fatty acid methyl ester (FAMEs) percentage (%) in the ground meal of various clean barley cultivars analyzed by GCMS.

FAME	AC Metcalfe	AAC Synergy	AAC Goldman	Lowe	CDC Copeland	CDC Mindon	CDC Bold	Harrington	CDC Bow	Line 1	Line 2	Line 3	Line 4	Line 5
C14:0	0.24±0.02e*	0.23±0.01e	0.24±0.0e	0.35±0.01d	0.23±0.0e	0.33±0.01d	0.36±0.06d	0.42±0.01bc	0.46±0.0ab	0.24±0.0e	0.48±0.01a	0.37±0.02cd	0.41±0.01bc	0.25±0.01e
C15:0	0.04±0.0h	0.05±0.0gh	0.07±0.01e-g	0.06±0.0f-h	0.06±0.01f-h	0.06±0.0f-h	0.08±0.02c-e	0.11±0.0a	0.10±0.0a-c	0.06±0.01gh	0.08±0.0d-f	0.09±0.01b-d	0.10±0.0a-c	0.04±0.0h
C16:0	26.0±0.31c-e	25.4±0.22d-g	25.0±0.19g-i	27.0±0.45ab	24.30±0.20ij	26.9±0.27ab	26.0±0.47c-e	25.2±0.13f-h	26.1±0.11cd	24.6±0.23h-j	26.2±0.05bc	24.0±0.18j	25.9±0.27c-f	27.1±0.09a
C16:1	0.06±0.01f	0.07±0.01f	0.08±0.01f	0.09±0.01ef	0.07±0.0f	0.08±0.01ef	0.12±0.03b-d	0.15±0.0a-c	0.12±0.0cd	0.08±0.0ef	0.11±0.0de	0.12±0.01b-d	0.15±0.01ab	0.07±0.01f
C18:0	1.23±0.12gh	1.37±0.01d-g	1.22±0.05gh	1.28±0.03f-h	1.30±0.02f-h	1.06±0.05h	1.31±0.11f-h	1.58±0.02b-e	1.83±0.08ab	1.14±0.01gh	1.53±0.03c-f	1.88±0.09a	1.75±0.05a-c	1.53±0.02c-f
C18:1 *t*	0.03±0.0b-d	0.03±0.01ab	0.04±0.01ab	0.04±0.01ab	0.02±0.0d	0.02±0.0d	0.02±0.0cd	0.04±0.0a	0.04±0.0ab	0.02±0.0d	0.04±0.0ab	0.03±0.0b-d	0.03±0.0bc	0.03±0.0b-d
C18:1 *c*	10.9±0.22f	10.7±0.40f	11.6±0.13c-f	10.6±1.98f	14.8±0.34a	11.3±0.22d-f	11.8±0.11b-f	13.2±0.18a-c	11.0±0.30f	11.7±0.13b-f	13.4±0.24ab	12.75±0.13b-e	13.0±0.18b-d	11.1±0.06ef
C18:2	53.2±0.70ab	53.6±0.26ab	53.7±0.26a	54.3±0.47a	51.8±0.07bc	53.3±0.03ab	51.3±1.72c	48.1±0.27d	48.7±0.59d	53.4±0.23ab	47.8±0.32d	48.3±0.81d	47.0±0.08d	52.9±0.31a-c
C20:0	0.09±0.02f	0.10±0.01ef	0.11±0.01ef	0.16±0.03bc	0.11±0.01d-f	0.10±0.01ef	0.15±0.02bc	0.22±0.01a	0.16±0.01bc	0.09±0.0f	0.13±0.0c-e	0.16±0.01bc	0.17±0.0b	0.11±0.01d-f
C20:1	0.40±0.03de	0.42±0.04de	0.43±0.02de	0.38±0.02e	0.44±0.02de	0.43±0.01de	0.53±0.07a-c	0.56±0.02ab	0.53±0.04a-c	0.49±0.01b-d	0.49±0.01b-d	0.60±0.03a	0.61±0.02a	0.42±0.02de
C18:3 α	4.3±0.05fg	4.7±0.23d-f	5.0±0.0b-e	4.3±0.22f-h	4.3±0.13f-h	4.1±0.28gh	4.5±0.39e-g	4.7±0.09d-f	4.9±0.15c-e	5.6±0.08ab	3.7±0.06h	5.4±0.28a-c	5.3±0.38a-d	4.2±0.13f-h
C20:2	0.03±0.01d	0.05±0.01d	0.04±0.0d	0.05±0.01d	0.04±0.0d	0.05±0.01cd	0.07±0.02bc	0.11±0.0a	0.11±0.01a	0.05±0.0d	0.09±0.0ab	0.11±0.01a	0.11±0.0a	0.05±0.01d
C22:0	0.08±0.02h	0.11±0.01f-h	0.11±0.0e-h	0.15±0.02cd	0.12±0.01d-g	0.10±0.0f-h	0.19±0.03b	0.26±0.0a	0.20±0.0b	0.09±0.0gh	0.14±0.01de	0.20±0.02b	0.19±0.0b	0.11±0.01d-h
C22:1	0.03±0.01e	0.04±0.01e	0.04±0.0de	0.04±0.01de	0.03±0.01e	0.05±0.01c-e	0.05±0.01c-e	0.07±0.01a-c	0.08±0.01a	0.05±0.0c-e	0.06±0.0b-d	0.08±0.01ab	0.08±0.01a	0.03±0.01e
C24:0	0.06±0.02fg	0.07±0.01e-g	0.06±0.0g	0.09±0.01e-g	0.08±0.01e-g	0.09±0.01ef	0.10±0.02e	0.19±0.01a	0.18±0.0ab	0.07±0.0e-g	0.14±0.01cd	0.16±0.01a-c	0.15±0.0b-d	0.08±0.01e-g
CATME	0.04±0.0c	0.04±0.0c	0.04±0.0c	0.03±0.01c	0.03±0.0c	0.04±0.01c	0.05±0.02bc	0.07±0.01ab	0.08±0.0a	0.04±0.0c	0.07±0.01ab	0.09±0.02a	0.08±0.01a	0.04±0.0c
CLnA	0.07±0.01e	0.08±0.0de	0.09±0.01de	0.08±0.01e	0.06±0.01e	0.07±0.01e	0.13±0.05bc	0.12±0.0b-d	0.16±0.0ab	0.09±0.01de	0.19±0.0a	0.15±0.01b	0.15±0.01b	0.09±0.0c-e
SFA	27.74	27.33	26.81	29.09	26.2	28.64	28.19	27.98	29.03	26.29	28.7	26.86	28.67	29.22
USFA	69.02	69.69	71.02	69.88	71.56	69.4	68.52	67.05	65.64	71.48	65.88	67.54	66.43	68.89
Ratio U/S	2.5	2.5	2.6	2.4	2.7	2.4	2.4	2.4	2.3	2.7	2.3	2.5	2.3	2.4
Other	0.04	0.04	0.04	0.03	0.03	0.04	0.05	0.07	0.08	0.04	0.07	0.09	0.08	0.04

*Results are the mean of three measurements ± SD. Mean values that do not share a letter in each row are significantly different at the 0.05 level using Tukey test. Statistical analysis was done using JMP 16 (SAS, NC, USA).

Methyl myristate (C14:0); Methyl pentadecanoate (C15:0); Methyl palmitate (C16:0); Methyl palmitoleate(C16:1); Methyl stearate (C18:0); trans-9-Elaidic acid methyl ester (C18:1t); cis-9-Oleic acid methyl ester (C18:1c); Methyl linoleate (C18:2); Methyl arachidate (C20:0); Methyl cis-11-eicosenoate (C20:1); Methyl linolenate (C18:3α); cis-11,14-Eicosadienoic acid methyl ester (C20:2); Methyl behenate (C22:0); Methyl erucate (C22:1); Methyl lignocerate (C24:0); Conjugated alpha linolenic acid (CLnA); saturated fatty acids (SFA); Unsaturated fatty acids (USFA); Ratio unsaturated fatty acids/saturated fatty acids (ratio U/S); Citric acid trimethyl ester (CATME)

A.

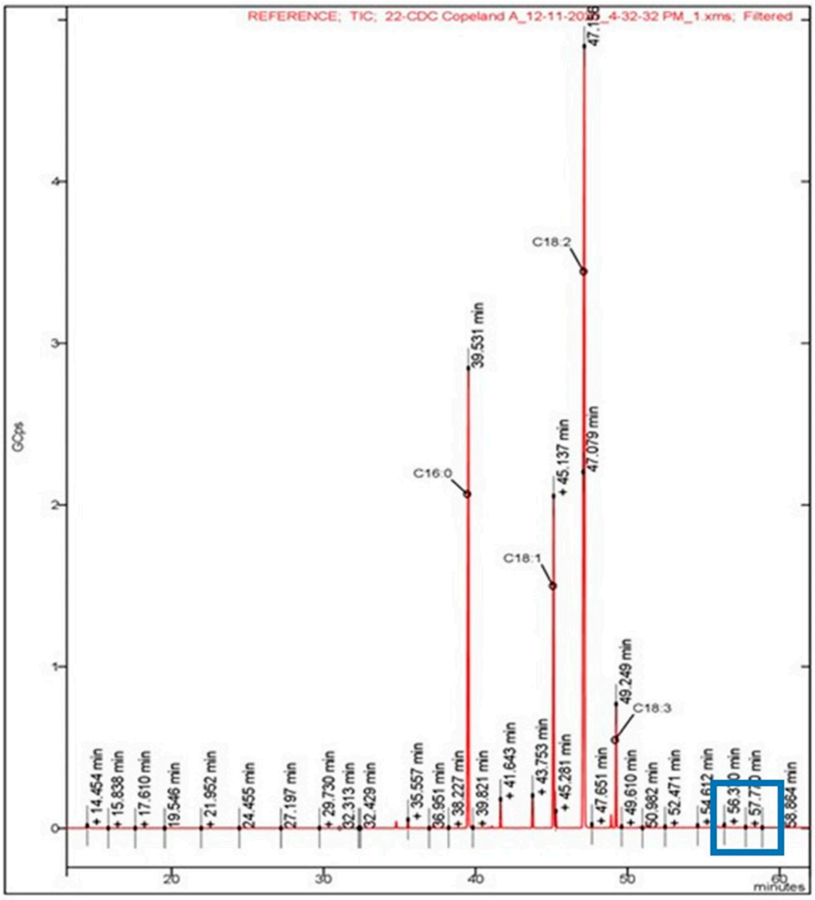

B.

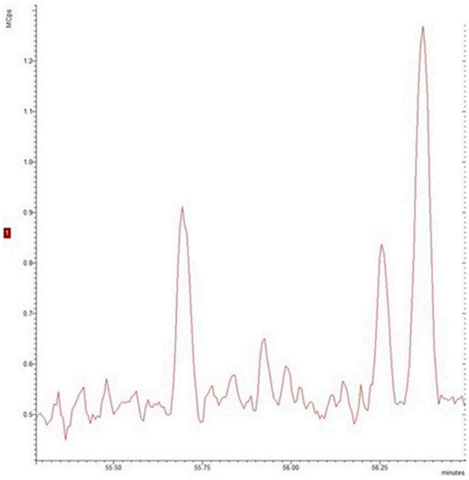

Supplementary Figure S1. GC/MS chromatogram of CDC Copeland grain sample showing the major peaks. C. Enlarged insert (blue square in A) of the chromatogram, Rt 55.69 is Citric acid trimethyl ester; Rt 56.261 is Methyl 9 cis, 11 trans, 13 trans-Octadecatrienoate.
